# Prevalence of hypothyroidism in older adults and its association with cognition: a cross-sectional study from a South Indian ageing urban cohort

**DOI:** 10.1093/braincomms/fcae391

**Published:** 2024-12-05

**Authors:** Angeline S Jessy, Sandhya G., Monisha S., Jonas S. Sundarakumar, Albert Stezin, Thomas Gregor Issac

**Affiliations:** Centre for Brain Research, Indian Institute of Science, Bengaluru 560012, India; Centre for Brain Research, Indian Institute of Science, Bengaluru 560012, India; Centre for Brain Research, Indian Institute of Science, Bengaluru 560012, India; Centre for Brain Research, Indian Institute of Science, Bengaluru 560012, India; Centre for Brain Research, Indian Institute of Science, Bengaluru 560012, India; Centre for Brain Research, Indian Institute of Science, Bengaluru 560012, India

**Keywords:** cognition, hypothyroidism, medication

## Abstract

The study conducted on a South Indian urban cohort aimed to emphasize the prevalence and patterns of hypothyroidism and its association with cognition among individuals aged 45 years and above. A cross-sectional design was adopted, utilizing data from the Tata Longitudinal Study of Aging cohort, comprising 1201 non-demented participants in Bangalore, South India. The study contains detailed clinical assessments, including medical history, physical examination and cognitive tests such as the COGNITO battery, Hindi Mental State Examination and Addenbrooke’s Cognition Examination III. Biochemical tests were utilized to quantify plasma levels of thyroid-stimulating hormone, triiodothyronine and thyroxine. Participants were categorized based on medication history and thyroid hormone levels. The study findings showed a 17.69% prevalence of hypothyroidism, with 6.22% being classified as overt hypothyroidism and 93.78% as subclinical hypothyroidism. The prevalence was significantly higher in females compared with males (*P* = 0.043). Individuals with hypothyroidism are more frequently diagnosed with mild cognitive impairment than people with euthyroid (*P* = 0.008). Furthermore, on the classification based on thyroid medication history, the ineffective treatment group performed poorer in Addenbrooke’s Cognition Examination III fluency (*P* = 0.006), auditory attention (*P* = 0.001) and form matching (*P* = 0.024) tasks compared with the adequately treated group. The partially treated group performed poorer in visual attention (*P* = 0.005) and vocabulary (*P* = 0.043) compared with the effectively treated group. The study identified a notable prevalence of hypothyroidism in the cohort, with females exhibiting a higher prevalence. Our study suggests that the timely management of thyroid disorders with medications is crucial not only to prevent hormonal imbalances but also to improve cognitive functioning.

## Teaser

This study aimed to identify the association of hypothyroidism with cognition. Jessy *et al*. showed a hypothyroidism prevalence of 17.69%. People with hypothyroidism were found to be more frequently diagnosed with mild cognitive impairment. The ineffectively treated hypothyroidism group had deficits in fluency, attention and visuospatial tasks.

## Graphical Abstract

**Graphical Abstract:**
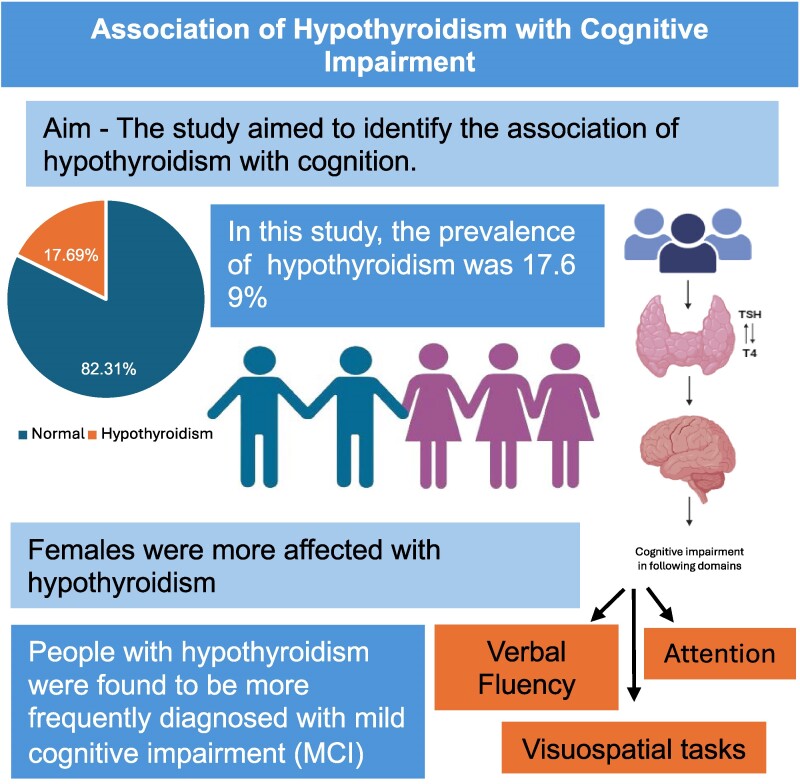

## Introduction

The thyroid gland plays a vital role in the development of neurodegenerative conditions and cognitive decline, which becomes pronounced with advancing age.^1^ Thyroid disorders are challenging to diagnose and treat, due to atypical presentations and the presence of a wide variety of comorbid conditions. Untreated thyroid dysfunction is associated with significant morbidity in the elderly.^2^

Thyroid disorders include hypothyroidism and hyperthyroidism. In hypothyroidism, the thyroid gland produces an insufficient amount of triiodothyronine (T3) and thyroxine (T4) hormones resulting in lethargy, weight gain, depression, abnormal bone development, cognitive impairment and hyporeflexia. On the other hand, hyperthyroidism increases the production of T3 and T4 hormones and manifests in symptoms like sweating, arrhythmia (irregular heartbeat), weight loss, nervousness, hyperreflexia and anxiety.

Thyroid disorders, particularly hypothyroidism, have a high prevalence. In India, a study conducted in eight major cities of the country in 2013 identified the prevalence of hypothyroidism to be 10.95%. The prevalence was found to be higher in females and the elderly population.^3^

Thyroid hormones play a key role in growth and neuronal development.^4^ Studies have shown that hypothyroidism is linked with cognitive impairment.^5-7^ Hypothyroidism is one of the causes of reversible dementia, meaning treatment of hypothyroidism can reverse the cognitive impairment caused.^8,9^ Hypothyroidism is associated with reduced functioning in several domains of cognition such as attention, memory, language, executive functioning and perceptuo-motor abilities.^10^

A significantly higher proportion of people with dementia were also found to have subclinical hypothyroidism than their cognitively healthy counterparts. This suggests that there is a link between subclinical hypothyroidism and cognitive decline.^11^ Individuals with subclinical hypothyroidism [elevated thyroid-stimulating hormone (TSH) levels] for a prolonged period have been found to develop cognitive impairment, especially in memory and verbal fluency-based tests.^12-14^ Lower T4 levels in elderly women are significantly associated with the risk of cognitive decline, especially in episodic memory.^15^ It is still unknown if subclinical hypothyroidism causes cognitive impairment in the elderly. Since it does not affect the quality of life of the individual, studies on treating subclinical hypothyroidism remain inconclusive.^16^

One of the major risk factors for cognitive decline and dementia is old age. Since the burden of hypothyroidism also increases with age, the risk of developing cognitive impairment is further increased due to the higher prevalence of hypothyroidism.^17^

In addition to studies that explore the impact of thyroid dysfunction on cognition, studies have shown that thyroid medication plays an important role in improving cognitive function.^9,18^ A meta-analysis by Uma *et al*.^19^ found that timely intervention and maintenance of optimal thyroid hormone levels through medications can improve memory, attention, language and visuospatial abilities.

Although many studies suggest thyroid disorders to be associated with cognitive performance, an individual participant data meta-analysis by van Vliet *et al*.^20^ shows no significant association between thyroid disorders and cognitive functioning. With such mixed evidence on the link between hypothyroidism and cognition, it becomes necessary for more cohort studies to investigate this association. The current study aims to identify the prevalence of hypothyroidism and to analyse the links with cognitive performance in older adults in an urban cohort of southern India.

## Materials and methods

### Study participants and study design

The present study follows a cross-sectional design. The study participants belong to the Tata Longitudinal Study of Ageing (TLSA) cohort consisting of non-demented participants ≥ 45 years of age residing in Bangalore, South India. Baseline data from the cohort were utilized for the current study. A total of 1201 participants who had completed data for basic socio-demographic characteristics and thyroid-related blood parameters such as TSH and T4 were included in the study.

### Clinical and cognitive assessments

All the participants underwent detailed clinical assessments wherein data about their clinical history, hypothyroidism medication history, depression [Geriatric Depression Scale (GDS)] and anxiety [Generalized Anxiety Disorder (GAD)] status were collected. The participants were also tested for their cognitive functioning using various tests including Hindi Mental State Examination (HMSE), Addenbrooke’s Cognitive Examination III (ACE III), Computerized Assessment of Adult Information Processing (COGNITO) neuropsychological battery and Clinical Dementia Rating (CDR).

HMSE is a cognitive screening tool used to assess orientation, registration, attention, recall and language. It is adapted from the Mini-Mental State Examination for the Indian illiterate population.^21^ The ACE III is a 100-point scale measuring the global cognitive function of five cognitive domains, namely attention and orientation, memory, verbal fluency, visuospatial abilities and language. The scale is culturally adapted for the Indian population for use in the English language in the present study.^22^

To test the participants’ cognitive function in various cognitive domains in detail, COGNITO battery was used. COGNITO battery is a computer-based neuropsychological battery that can detect subtle changes in cognition. The tests of the COGNITO battery are as follows: attention (reaction time, auditory attention and visual attention), language (reading and sentence comprehension, word comprehension, semantic associations, categorical fluency, letter fluency and vocabulary), memory (episodic memory immediate and delayed recall, name–face recognition and implicit memory) and visuospatial abilities (visuospatial span, form matching, Stroop test and construction ability). We used an adapted version of COGNITO battery that has been validated in the Indian population.^23^ The CDR is a clinician-administered scale that is used to assess an individual in three cognitive (memory, orientation, judgment and problem-solving) and three functional domains (community affairs, home and hobbies and personal care). Each domain is rated on a 5-point scale, with personal care scored on a 4-point scale. Through a comprehensive review, a CDR score of 0.5 was used to classify participants with mild cognitive impairment.^24^

### Biochemical tests

The categorization of participants into groups based on thyroid function was performed using biochemical tests. For that purpose, fasting blood samples were collected from the study participants by a phlebotomist. From each individual, 5 ml of collected blood was used for biochemical investigation. Blood analysis was done at RV Metropolis Laboratory in Malleshwaram, Bengaluru. The laboratory is accredited by the National Accreditation Board for Testing Calibration Laboratories in India.

### Diagnosis of hypothyroidism

Thyroid hormones were quantified using chemiluminescence immunoassays. TSH and T4 levels were utilized to classify individuals with thyroid disorders. Normal reference ranges were defined as TSH levels between 0.54 and 5.30 mIU/mL and T4 levels between 5.1 and 14.1 µg/dL.^25^ Participants having TSH levels above the normal range were categorized as hypothyroid. They were further categorized into subclinical hypothyroidism and overt hypothyroidism groups based on TSH and T4 levels. Participants with elevated TSH levels and normal T4 levels were classified as subclinical hypothyroidism, while those with elevated TSH and low T4 levels were classified as overt hypothyroidism.^26^

Considering both medication history and serum levels of TSH and T4, the participants were classified into four groups: normal (no medications and normal TSH levels), undetected hypothyroidism (no medications and high TSH levels), inadequately treated hypothyroidism (medication taken and high TSH levels) and adequately treated hypothyroidism (medication taken and normal TSH levels). Furthermore, the inadequately treated group was classified into partially adequate (medication taken, high TSH levels and normal T4 levels) and ineffective (medication taken, high TSH levels and low T4 levels) treatment groups.^27^

### Statistical analysis

Mann–Whitney *U* test was performed to compare mean differences in age, education, HMSE, ACE III, GAD, GDS and COGNITO scores, among two groups. A *χ*^2^ test was performed to find the association of gender, comorbidities and CDR among the groups. A generalized linear model was used to find the relationship between cognitive scores and hypothyroidism. Model 1 was unadjusted, Model 2 was adjusted for age, gender and years of education, and Model 3 was adjusted for age, gender, years of education and Apoε4 status. The covariates were chosen since all four of them can have an independent influence on the outcome variable, cognition. IBM Statistical Package for Social Sciences software version 28.0.1.1(15) has been used for statistical analysis.

## Results

**Figure 1:**
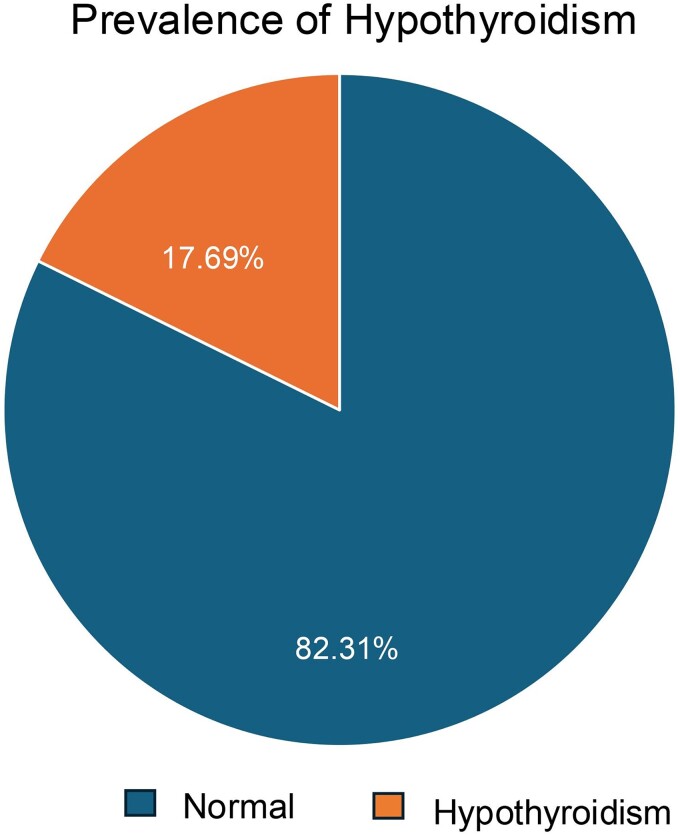
**Prevalence of hypothyroidism in the TLSA cohort.** This figure illustrates the prevalence of hypothyroidism in the TLSA cohort. The total sample size is *N* = 1181, with 209 (17.69%) participants diagnosed with hypothyroidism and 972 (82.30%) participants classified as euthyroid.

A total of 1201 participants were included in the study, among which 209 (17.40%) had hypothyroidism identified based on TSH levels (Fig. 1). There were 13 individuals with overt hypothyroidism (1.08%) and 196 individuals with subclinical hypothyroidism (16.32%). After excluding 20 individuals with hyperthyroidism, a total of 1181 participants were considered for the study. The mean age of the participants included was 62.36 ± 9.456, and 52.6% of the subjects were male, whereas 47.4% were female.

Females had a significantly higher prevalence of hypothyroidism than males (*P* = 0.043). Hypothyroidism had a significant association with height (*P* = 0.002) and weight (*P* = 0.002). The hypothyroidism group had significantly higher total cholesterol (*P* = 0.007), triglycerides (*P* = 0.044), low-density lipoprotein (*P* = 0.026) and very low-density lipoprotein (*P* = 0.032) levels than the normal (euthyroid) group. Those with hypothyroidism were found to have significantly higher low-density lipoprotein/high-density lipoprotein ratios when compared with those who were euthyroid (*P* = 0.043). Diabetes mellitus was also found to be significantly associated with hypothyroidism (*P* = 0.029). When comparing the GAD and GDS scores, no difference was found between the hypothyroid and euthyroid (Table 1).

**Table 1:** Comparison of demographic characteristics between euthyroid and hypothyroidism

	Euthyroid	Hypothyroidism	*P*-value
Age	62 (55, 68.74)	63 (56, 70)	0.363
Gender			
Male	52.60%	44.50%	
Female	47.40%	55.50%	0.034^*^
CDR			
0	92%	86.10%	
0.5	8%	13.90%	0.008^*^
Height	161 (154.5, 168)	159 (152, 166.25)	0.002^*^
Weight	70 (62, 79.28)	66.3 (60.3, 74.62)	0.002^*^
Education	15 (13, 17)	15 (13, 17)	0.231
Hypertension	67.30%	60.80%	0.071
Diabetes	37.70%	29.70%	0.029^*^
Dyslipidaemia	89.10%	90.90%	0.439
Cholesterol	185.5 (154.5, 216.5)	198 (162.55, 223.05)	0.007^*^
Triglycerides	132.4 (96.3, 177.8)	137 (105, 187.5)	0.044^*^
HDL	44 (37.9, 51.9)	44 (38.55, 52.2)	0.652
LDL	116.1 (89.3, 142.7)	125 (95.75, 147.9)	0.026^*^
VLDL	25.9 (19, 35.3)	27 (20.95, 37.3)	0.032^*^
Cholesterol/HDL ratio	4.1 (3.4, 5)	4.3 (3.5, 5.1)	0.092
LDL/HDL ratio	2.6 (2, 3.3)	2.9 (2.1, 3.5)	0.043^*^
Glycosylated haemoglobin	6.2 (5.8, 7.2)	6.15 (5.7, 7)	0.145
Homocysteine	16.86 (12.87, 22.78)	18.44 (13.13, 23.27)	0.157
GDS	1 (0, 4)	1 (0,4)	0.220
GAD	0 (0, 0)	0 (0, 0)	0.562
HMSE	31 (30,31)	31 (30,31)	0.584
ACE total	93 (89, 96)	93 (89, 96)	0.844

ACE III, Addenbrooke’s Cognitive Examination III; CDR, Clinical Dementia Rating; GAD, Generalized Anxiety disorder; GDS, Geriatric Depression Scale; HDL, high-density lipoprotein; HMSE, Hindi Mental State Examination; LDL, low-density lipoprotein; VLDL, very low-density lipoprotein.

^*^Significant at *P* < 0.05.

Diagnosis of mild cognitive impairment and hypothyroidism was found to be significantly associated with each other (*P* = 0.008). Although people with hypothyroidism had a higher chance of being diagnosed with mild cognitive impairment, there was no significant difference in scores of HMSE, ACE III and tests of COGNITO battery between the hypothyroid and euthyroid groups. Generalized linear model was used, with hypothyroidism-based category as the independent variable and cognition assessed by HMSE, ACE III and COGNITO as dependent variables. There was no association found between hypothyroidism and any cognitive test in all three models (Table 2).

**Table 2:** Comparison of cognitive function and performance measures between euthyroid and hypothyroidism groups

	Model 1		Model 2		Model 3	
	*β* (95% CI)	*P*-value	*β* (95% CI)	*P*-value	*β* (95% CI)	*P*-value
HMSE	−0.086 (−0.247, 0.075)	0.294	−0.079 (−0.238, 0.080)	0.330	−0.049 (−0.224, 0.126)	0.581
ACE total	−0.02 (−1.035, 0.995)	0.969	−0.174 (−1.094, 0.746)	0.711	−0.342 (−1.339, 0.656)	0.502
ACE attention	0.083 (−0.133, 0.299)	0.451	0.088 (−0.117, 0.293)	0.401	0.059 (−0.160, 0.279)	0.597
ACE memory	−0.189 (−0.607, 0.230)	0.377	−0.229 (−0.628, 0.169)	0.259	−0.311 (−0.746, 0.124)	0.161
ACE fluency	0.099 (−0.210, 0.409)	0.529	−0.007 (−0.294, 0.281)	0.963	0.002 (−0.303, 0.307)	0.990
ACE language	−0.066 (−0.317, 0.184)	0.604	−0.077 (−0.323, 0.168)	0.536	−0.119 (−0.373, 0.135)	0.358
ACE visuospatial	0.053 (−0.211, 0.316)	0.696	0.052 (−0.195, 0.299)	0.680	0.027 (−0.245, 0.300)	0.845
Reaction time	0.41 (−6.584, 7.403)	0.909	−0.333 (−7.155, 6.489)	0.924	2.936 (−4.450, 10.323)	0.436
Reading and sentence comprehension	−0.108 (−0.285, 0.07)	0.236	−0.106 (−0.279, 0.067)	0.231	−0.182 (−0.365, 0.001)	0.052
Auditory attention	−0.104 (−0.293, 0.085)	0.280	−0.095 (−0.282, 0.092)	0.319	−0.160 (−0.354, 0.034)	0.105
Visual attention	−0.239 (−0.537, 0.059)	0.117	−0.195 (−0.476, 0.086)	0.175	−0.282 (−0.585, 0.020)	0.067
Stroop test	0.05 (−0.453, 0.553)	0.846	0.062 (−0.429, 0.552)	0.805	0.138 (−0.392, 0.668)	0.609
Episodic memory IR	−0.094 (−0.328, 0.139)	0.428	−0.139 (−0.360, 0.082)	0.217	−0.163 (−0.405, 0.080)	0.188
Episodic memory DR	−0.01 (−0.345, 0.325)	0.954	−0.046 (−0.360, 0.269)	0.775	−0.093 (−0.433, 0.248)	0.594
Visuospatial span	−0.014 (−0.232, 0.203)	0.898	−0.005 (−0.219,0.209)	0.963	−0.048 (−0.281, 0.186)	0.688
Form matching	−0.086 (−0.361, 0.190)	0.541	−0.081 (−0.348, 0.187)	0.555	−0.197 (−0.486, 0.092)	0.182
Word comprehension	−0.03 (−0.213, 0.153)	0.746	−0.042 (−0.221, 0.137)	0.648	−0.019 (−0.215, 0.176)	0.846
Semantic association	−0.003 (−0.119, 0.114)	0.966	−0.001 (−0.114, 0.112)	0.988	0.044 (−0.077, 0.515)	0.473
Name–face recognition	−0.172 (−0.562, 0.218)	0.388	−0.195 (−0.559, 0.169)	0.293	−0.140 (−0.531, 0.252)	0.483
Categorical fluency	0.41 (−0.572, 1.393)	0.413	0.216 (−0.677, 1.110)	0.635	−0.071 (−1.006, 0.864)	0.882
Letter fluency	0.419 (−0.474, 1.313)	0.538	0.267 (−0.589, 1.123)	0.541	−0.133 (−1.036, 0.771)	0.773
Vocabulary	−0.119 (−1.901, 1.663)	0.896	−0.216 (−1.960, 1.528)	0.808	−0.089 (−1.962, 1.783)	0.926
Construction ability	1.642 (−0.375, 3.660)	0.111	1.473 (−0.514, 3.461)	0.146	0.808 (−1.261, 2.878)	0.444
Implicit memory	0.059 (−0.117, 0.234)	0.513	0.051 (−0122, 0.225)	0.562	0.064 (−0.118, 0.246)	0.492

Model 1: not adjusted for any covariate. Model 2: adjusted age, gender and education. Model 3: Model 2 + ApoE.

ACE III, Addenbrooke’s Cognitive Examination III; DR, delayed recall; IR, immediate recall; HMSE, Hindi Mental State Examination.

Furthermore, analysis was performed after excluding individuals with overt hypothyroidism. Visual attention scores were found to be lesser in the subclinical hypothyroidism group than the euthyroid in Model 3 {*β* [95% confidence interval (CI)] = −0.338 [−0.649, −0.027], *P* = 0.033} (Table 3).

**Table 3:** Comparison of cognitive function and performance measures between subclinical hypothyroidism and euthyroid groups

	Model 1		Model 2		Model 3	
	*β* (95% CI)	*P*-value	*β* (95% CI)	*P*-value	*β* (95% CI)	*P*-value
HMSE	−0.093 (−0.259, 0.072)	0.270	−0.082 (−0.246, 0.082)	0.326	−0.053 (−0.233, 0.126)	0.562
ACE total	−0.086 (−1.129, 0.957)	0.872	−0.184 (−1.132, 0.763)	0.703	−0.344 (−1.369, 0.681)	0.511
ACE attention	0.058 (−0164, 0.281)	0.608	0.066 (−0.145, 0.277)	0.542	0.036 (−0.190, 0.261)	0.756
ACE memory	−0.202 (−0.632, 0.229)	0.358	−0.222 (−0.633, 0.188)	0.288	−0.301 (0.748, 0.145)	0.186
ACE fluency	0.136 (−0.181, 0.452)	0.400	0.043 (−0.251, 0.338)	0.772	0.064 (−0.249, 0.377)	0.689
ACE language	−0.098 (−0.356, 0.160)	0.458	−0.100 (−0.353, 0.153)	0.439	−0.140 (−0.401, 0.120)	0.291
ACE visuospatial	0.019 (−0.251, 0.290)	0.889	0.029 (−0.226, 0.283)	0.825	−0.001 (−0.281, 0.278)	0.992
Reaction time	−0.677 (−7.846, 6.492)	0.853	−1.643 (−8.639, 5.352)	0.645	1.454 (−6.128, 9.037)	0.707
Reading and sentence comprehension	−0.088 (−0.271, 0.094)	0.344	−0.082 (−0.259, 0.095)	0.365	−0.157 (−0.346, 0.031)	0.102
Auditory attention	−0.075 (−0.263, 0.113)	0.433	−0.063 (−0.249, 0.123)	0.509	−0.125 (−0.325, 0.075)	0.220
Visual attention	−0.294 (−0.601, 0.013)	0.061	−0.239 (−0.529, 0.050)	0.105	−0.338 (−0.649, −0.027)	0.033^*^
Stroop test	0.052 (−0.464, 0.569)	0.842	0.051 (−0.453, 0.555)	0.842	0.135 (−0.412, 0.682)	0.628
Episodic memory IR	−0.087 (−0.328, 0.154)	0.477	−0.124 (−0.352, 0.103)	0.285	−0.148 (−0.399, 0.103)	0.247
Episodic memory DR	−0.003 (−0.347, 0.342)	0.988	−0.026 (−0350, 0.299)	0.877	−0.075 (−0.428, 0.278)	0.677
Visuospatial span	−0.014 (−0.238, 0.209)	0.901	−0.001 (−0.221, 0.220)	0.995	−0.049 (−0.290, 0.192)	0.690
Form matching	−0.088 (−0.371, 0.194)	0.540	−0.077 (−0.351, 0.198)	0.584	−0.197 (−0.496, 0.102)	0.197
Word comprehension	−0.034 (−0.224, 0.156)	0.725	−0.044 (−0.230, 0.142)	0.643	−0.018 (−0.222, 0.185)	0.861
Semantic association	0.007 (−0.113, 0.126)	0.913	0.011 (−0.105, 0.128)	0.850	0.061 (−0.064, 0.186)	0.339
Name–face recognition	−0.196 (−0.599, 0.206)	0.339	−0.198 (−0.573, 0.176)	0.300	−0.147 (−0.551, 0.258)	0.478
Categorical fluency	0.390 (−0.627, 1.407)	0.452	0.237 (−0.690, 1.164)	0.617	−0.065 (−1.037, 0.908)	0.897
Letter fluency	0.576 (−0.349, 1.501)	0.222	0.437 (−0.450, 1.323)	0.335	0.035 (−0.903, 0.973)	0.942
Vocabulary	−0.395 (−2.225, 1.435)	0.672	−0.418 (−2.209, 1.372)	0.647	−0.294 (−2.216, 1.628)	0.764
Construction ability	1.812 (−0.266, 3.890)	0.088	1.635 (−0.411, 3.682)	0.117	1.006 (−1.120, 3.131)	0.354
Implicit memory	0.062 (−0.117, 0.242)	0.497	0.057 (−0.121, 0.235)	0.529	0.070 (−0.116, 0.256)	0.460

Model 1: not adjusted for any covariate. Model 2: adjusted age, gender and education. Model 3: model 2 + ApoE.

ACE III, Addenbrooke’s Cognitive Examination III; DR, delayed recall; IR, immediate recall; HMSE, Hindi Mental State Examination.

^*^Significant at *P* < 0.05.

Subsequently, a sex-stratified analysis was performed. In the unadjusted model, males with hypothyroidism were found to have significantly lower scores in ACE memory [*β* (95% CI) = −0.635 (−1.224, −0.046), *P* = 0.035] and name–face recognition [*β* (95% CI) = −0.597 (−1.169, −0.026), *P* = 0.040] tasks as compared with euthyroid males. Such a difference was not observed in females. In the model adjusted for age and education, males with hypothyroidism showed significantly poorer performance in ACE memory [*β* (95% CI) = −0.585 (−1.143, −0.027), *P* = 0.040] and name–face recognition [*β* (95% CI) = −0.557 (−1.088, −0.026), *P* = 0.040] compared with euthyroid males (Table 4). However, we did not find any significant difference in hypothyroid females when compared with euthyroid females. Similarly, when adjusted for age, education and Apoε4 status, males with hypothyroidism showed significantly lower scores on ACE memory [*β* (95% CI) = −0.723 (−1.329, −0.118), *P* = 0.019], reading and sentence comprehension [*β* (95% CI) = −0.294 (−0.567, −0.020), *P* = 0.035] and form matching [*β* (95% CI) = −0.435 (−0.844, −0.025), *P* = 0.038] tasks as compared with euthyroid males. Females with hypothyroidism showed poorer performance in auditory attention [*β* (95% CI) = −0.361 (−0.654, −0.069), *P* = 0.016] and visual attention [*β* (95% CI) = −0.510 (−0.971, −0.048), *P* = 0.030] tasks than euthyroid females (Table 5).

**Table 4:** Comparison of cognitive function and performance measures between hypothyroidism and euthyroid group in males

	Model - 1		Model- 2		Model – 3	
	*β* (95% CI)	*P*-value	*β* (95% CI)	*P*-value	*β* (95% CI)	*P*-value
HMSE	−0.028 (−0.261, 0.205)	0.816	0.002 (−0.228, 0.231)	0.988	0.065 (−0.187, 0316)	0.615
ACE total	−0.099 (−1.493, 1.294)	0.889	−0.003 (−1.286, 1.280)	0.996	−0.209 (−1.596, 1.178)	0.768
ACE attention	0.137 (−0.134, 0.408)	0.323	0.141 (−0.125, 0.406)	0.299	0.127 (−0.149, 0.404)	0.367
ACE memory	−0.635 (−1.224, −0.046)	0.035^*^	−0.585 (−1.143, −0.027)	0.040^*^	−0.723 (−1.329, −0.118)	0.019^*^
ACE fluency	0.141 (−0.318, 0.601)	0.546	0.132 (−0.305, 0.570)	0.533	0.158 (−0.303, 0.620)	0.501
ACE language	0.038 (−0.331, 0.408)	0.839	0.084 (−0.0274, 0.442)	0.644	0.020 (−0.348, 0.387)	0.917
ACE visuospatial	0.219 (−0.126, 0.565)	0.214	0.224 (−0.112, 0.561)	0.191	0.209 (−0.158, 1.247)	0.264
Reaction time	−0.384 (−11.003, 10.235)	0.944	−0.600 (−11.062, 9.863)	0.911	0.428 (−10.712, 11.568)	0.940
Reading and sentence comprehension	−0.203 (−0.463, 0.058)	0.127	−0.199 (−0.455, 0.056)	0.127	−0.294 (−0.567, 4.433)	0.035^*^
Auditory attention	0.072 (−0.182, 0.327)	0.576	0.081 (−0.172, 0.334)	0.532	0.076 (−0.174, 0.327)	0.550
Visual attention	−0.046 (−0.433, 0.341)	0.816	−0.016 (−0.386, 0.355)	0.934	−0.048 (−0.432, 0.337)	0.808
Stroop test	0.259 (−0.544, 1.061)	0.528	0.245 (−0.548, 1.038)	0.545	0.232 (−0.660, 1.123)	0.610
Episodic memory IR	−0.262 (−0.587, 0.063)	0.114	−0.257 (−0.578, 0.063)	0.115	−0.270 (−0.615, 0.074)	0.124
Episodic memory DR	−0.460 (−0.937, 0.016)	0.058	−0.442 (−0.910, 0.026)	0.064	−0.418 (−0.920, 0.085)	0.103
Visuospatial span	−0.117 (−0.451, 0.216)	0.490	−0.104 (−0.434, 0.225)	0.535	−0.207 (−0.561, 0.148)	0.253
Form matching	−0.237 (−0.638, 0.165)	0.248	−0.221 (−0.611, 0.170)	0.268	−0.435 (−0.844, −0.025)	0.038^*^
Word comprehension	0.043 (−0.231, 0.317)	0.760	0.019 (−0.251, 0.289)	0.889	0.047 (−0.227, 0.320)	0.738
Semantic associations	0.014 (−0.138, 0.166)	0.854	0.017 (−0.130, 0.164)	0.818	0.039 (−0.119, 0.198)	0.627
Fluid reasoning	−0.623 (−4.872, 3.625)	0.774	−1.477 (−5.027, 2.074)	0.415	−1.099 (−4.636, 2.438)	0.543
Name–face recognition	−0.597 (−1.169, −0.026)	0.040^*^	−0.557 (−1.088, −0.026)	0.040^*^	−0.454 (−1.024, 0.117)	0.119
Categorical fluency	0.133 (−1.151, 1.416)	0.839	0.136 (−1.131, 1.403)	0.833	−0.096 (−1.423, 1.232)	0.888
Letter fluency	−0.176 (−1.445, 1.093)	0.786	−0.219 (−1.460, 1.022)	0.729	−0.670 (−1.963, 0.624)	0.310
Vocabulary	0.596 (−2.159, 3.350)	0.672	0.473 (−2.183, 3.128)	0.727	0.691 (−2.069, 3.451)	0.624
Construction ability	1.112 (−1.1468, 3.692)	0.398	0.882 (−1.668, 3.434)	0.498	0.308 (−2.231, 2.848)	0.812
Implicit memory	−0.041 (−0.312, 0.230)	0.767	−0.036 (−0.305, 0.232)	0.790	−0.010 (−0.289, 0.268)	0.943

Model 1: not adjusted for any covariate. Model 2: adjusted age and education. Model 3: Model 2 + ApoE.

ACE III, Addenbrooke’s Cognitive Examination III; DR, delayed recall; IR, immediate recall; HMSE, Hindi Mental State Examination.

^*^Significant at *P* < 0.05.

**Table 5:** Comparison of cognitive function and performance measures between hypothyroidism and euthyroid group in Females

	Model - 1		Model- 2		Model – 3	
	*β* (95% CI)	*P*-value	*β* (95% CI)	*P*-value	*β* (95% CI)	*P*-value
HMSE	−0.120 (−0.342, 0.102)	0.290	−0.148 (−0.368, 0.071)	0.988	−0.159 (−0.399, 0.081)	0.195
ACE total	0.124 (−1.352, 1.601)	0.869	−0.340 (−1.638, 0.958)	0.608	−0.606 (−2.014, 0.802)	0.399
ACE attention	0.117 (−0.209, 0.443)	0.482	0.036 (−0.270, 0.341)	0.819	−0.041 (−0.372, 0.289)	0.806
ACE memory	0.195 (−0.400, 0.790)	0.521	0.076 (−0.488, 0.640)	0.792	0.022 (−0.598, 0.643)	0.943
ACE fluency	0.005 (−0.411, 0.422)	0.980	−0.126 (−0.499, 0.246)	0.507	−0.165 (−0.563, 0.233)	0.416
ACE language	−0.172 (−0.514, 0.170)	0.325	−0.209 (−0.545, 0.127)	0.223	−0.242 (−0.592, 0.109)	0.117
ACE visuospatial	−0.021 (−0.413, 0.370)	0.915	−0.117 (−0.474, 0.240)	0.522	−0.180 (−0.577, 0.216)	0.373
Reaction time	0.120 (−8.990, 9.231)	0.979	−0.078 (−8.974, 8.818)	0.986	4.860 (−4.947, 14.666)	0.331
Reading and sentence comprehension	−0.029 (−0.273, 0.215)	0.816	−0.027 (−0.261, 0.206)	0.818	−0.100 (−0.344, 0.145)	0.424
Auditory attention	−0.243 (−0.520, 0.035)	0.087	−0.245 (−0.517, 0.027)	0.078	−0.361 (−0.654, −0.069)	0.016^*^
Visual attention	−0.352 (−0.797, 0.092)	0.120	−0.349 (−0.766, 0.068)	0.101	−0.510 (−0.971, −0.048)	0.030^*^
Stroop test	−0.103 (−0.728, 0.522)	0.747	−0.094 (−0.689, 0.500)	0.756	0.068 (−0.525, 0.661)	0.822
Episodic memory IR	−0.029 (−0.342, 0.284)	0.855	−0.035 (−0.339, 0.268)	0.820	−0.079 (−0.416, 0.261)	0.649
Episodic memory DR	0.340 (−0.088, 0.768)	0.120	0.292 (−0.124, 0.708)	0.169	0.172 (−0.283, 0.626)	0.459
Visuospatial span	0.083 (−0.198, 0.365)	0.562	0.084 (0.192, 0.361)	0.550	0.099 (−0.204, 0.403)	0.521
Form matching	0.054 (−0.325, 0.432)	0.782	0.048 (−0.318, 0.413)	0.798	0.008 (−0.396, 0.412)	0.969
Word comprehension	−0.092 (−0.336, 0.155)	0.470	−0.090 (−0.329, 0.148)	0.457	−0.084 (−0.364, 0.196)	0.557
Semantic associations	−0.014 (−0.188, 0.160)	0.874	−0.015 (−0.186, 0.155)	0.860	0.041 (−0.142, 0.223)	0.661
Fluid reasoning	−2.727 (−6.724, 1.270)	0.181	−0.938 (−4.593, 2.717)	0.615	−0.883 (−4.290, 2.524)	0.612
Name–face recognition	0.145 (−0.369, 0.658)	0.581	0.117 (−0.375, 0.609)	0.641	0.111 (−0.421, 0.643)	0.683
Categorical fluency	0.296 (−0.961,1.553)	0.644	0.268 (−0.988, 1.524)	0.676	0.016 (−1.297, 1.329)	0.981
Letter fluency	0.826 (−0.415, 2.068)	0.192	0.645 (−0.531, 1.821)	0.232	0.304 (−0.951, 1.559)	0.635
Vocabulary	−0.796 (−3.128, 1.535)	0.503	−0.764 (−3.056, 1.529)	0.514	−0.558 (−3.097, 1.980)	0.667
Construction ability	1.865 (−1.198, 4.927)	0.233	1.971 (−1.032, 4.973)	0.198	1.105 (−2.065, 4.274)	0.495
Implicit memory	0.122 (−0.107, 0.352)	0.297	0.117 (−0.110, 0.344)	0.311	0.129 (−0.111, 0.369)	0.293

Model 1: not adjusted for any covariate. Model 2: adjusted age and education. Model 3: Model 2 + ApoE.

ACE III, Addenbrooke’s Cognitive Examination III; DR, delayed recall; IR, immediate recall; HMSE, Hindi Mental State Examination.

^*^Significant at *P* < 0.05.

On comparing the four groups classified based on medication history and serum levels of TSH and T4 (normal, undetected, inadequately treated and adequately treated), the unadjusted Model 1 found that people with adequate treatment had better ACE fluency scores than the normal group [*β* (95% CI) = 0.568 (0.098, 1.039), *P* = 0.018] (Supplementary Table 1). It was also found that people with undetected hypothyroidism performed poorer in the name–face recognition task than the normal group [*β* (95% CI) = −0.602 (−1.123, −0.082), *P* = 0.017] (Supplementary Table 2). From Model 2, it was seen that people on adequate medication had better scores in the ACE fluency subcategory when compared with the normal group [*β* (95% CI) = 0.505 (0.063, 0.948), *P* = 0.025]. Additionally, after adjusting with Apoε4 status (Model 3), the inadequately treated group performed poorer in auditory attention [*β* (95% CI) = −0.436 (−0.827, −0.045), *P* = 0.029] and visual attention [*β* (95% CI) = −0.617 (−1.202, −0.032), *P* = 0.039] tasks when compared with the normal group (Supplementary Table 3).

On dividing the people on medication into three groups (adequate, partial and ineffective), Model 1 showed that the ineffective group had significantly lesser scores in ACE fluency [*β* (95% CI) = −2.435 (−4.171, −0.698), *P* = 0.006], auditory attention [*β* (95% CI) = −2.113 (−3.347, −0.880), *P* = 0.001] and form matching [*β* (95% CI) = −1.800 (−3.363, −0.237), *P* = 0.024] tasks than the adequately treated group (Supplementary Table 4). Also, the partially treated group had significantly lower scores in the visual attention task [*β* (95% CI) = −0.897 (−1.639, −0.155), *P* = 0.018] when compared with the adequately treated group. However, in the semantic association [*β* (95% CI) = 0.316 (90.051, 0.582), *P* = 0.019] and construction ability tasks [*β* (95% CI) = 3.931 (0.742, 7.121), *P* = 0.016], the partially treated group performed better than the adequately treated group (Supplementary Table 5). After adjusting for age, gender and years of education, it was found that the ineffective treatment group had significantly lower scores in ACE fluency [*β* (95% CI) = −2.173 (−3.760, −0.586), *P* = 0.007], auditory attention [*β* (95% CI) = −1.971 (−3.211, −0.731), *P* = 0.002] and form matching [*β* (95% CI) = −1.568 (−3.054, −0.082), *P* = 0.039] tasks than the adequately treated group. Also, the partially treated group performed better in construction ability task [*β* (95% CI) = 3.826 (0.602, 7.051), *P* = 0.020] but poorer in visual attention [*β* (95% CI) = −0.990 (−1.677, −0.303), *P* = 0.005] and vocabulary [*β* (95% CI) = −3.521 (−6.936, −0.107), *P* = 0.043] tasks when compared with the individuals with adequate treatment. In Model 3, ACE fluency [*β* (95% CI) = − 2.308 (−3.900, −0.716), *P* = 0.004], auditory attention [*β* (95% CI) = −1.979 (−3.01, −0.657), *P* = 0.003] and form matching [*β* (95% CI) = −1.546 (−3.024, −0.069), *P* = 0.040] scores were lower in the ineffective than adequately treated group. In the partially treated group, scores in visual attention [*β* (95% CI) = −1.056 (−1.820, −0.91), *P* = 0.007] and vocabulary tasks [*β* (95% CI) = −4.528 (−8.148, −0.907), *P* = 0.014] were lower in comparison with the adequately treated group.

## Discussion

The present study identified a 17.69% prevalence of hypothyroidism in an urban aging cohort from southern India. A previous study by Unnikrishnan *et al*.^3^ reported a 10.95% prevalence of overt hypothyroidism whereas our study identified a 1.1% prevalence of overt hypothyroidism. In our study, females had a higher prevalence of hypothyroidism than males, which is in accordance with the previous results.^28^

Individuals who had hypothyroidism were more likely to be diagnosed with mild cognitive impairment compared with individuals with normal thyroid function. This adds to previous evidence that suggests that people with hypothyroidism have a higher risk of developing dementia.^29^ The observed association with CDR scores can suggest that hypothyroidism may affect the functional domains of a person but let the cognition remain intact, causing a resultant increase in CDR scores leaving the neuropsychological test scores normal.

The study found significantly higher levels of several lipid parameters in people with hypothyroidism compared with normal. Evidence from previous studies indicates that hypothyroidism by itself can be a cause of dyslipidaemia.^30^ Binding of T4 and TSH with their respective receptors can alter the expression of genes involved in lipid metabolism. Thus, altering T4 and TSH concentrations affects the metabolism of lipids resulting in dyslipidaemia.^31^

Even in those individuals with subclinical hypothyroidism, the study found impairment in visual attention-based task compared with the euthyroid group. This suggests that treatment of subclinical hypothyroidism is necessary to maintain healthy cognition among the elderly.

Our results show that males with hypothyroidism had a significantly poorer performance in memory-related tasks in comparison with the euthyroid participants. The final adjusted Model 3 showed that males with hypothyroidism had impairments in memory, language and visuospatial abilities domains while females with hypothyroidism had impairments only in the attention domain. Sex-based differences in hypothyroidism cognitive impairment have not been studied widely. However, previous literature shows a higher prevalence of hypothyroidism in females, but higher mortality in males with hypothyroidism in a middle-aged cohort.^32^ Since males with hypothyroidism experience more symptoms, our study might have observed impairments in multiple domains in males with hypothyroidism. It is also to be noted that females with hypothyroidism are not spared from cognitive disturbances. A previous study by Eslami-Amirabadi *et al*.^33^ found that majority of females with hypothyroidism experience impairment in at least one cognitive domain with attention being one of the commonly affected domains. Our initial results on the sex-specific differences between the males and females provide the basis for further studies focussing on the reason for differential involvement of various cognitive domains.

Furthermore, we also considered the influence of thyroid medication on cognition. Adequate treatment corresponded with better ACE fluency scores, emphasizing the importance of optimal thyroid hormone levels for normal cognitive function when compared with ineffectively treated group. This aligns with the existing literature that shows that adequate treatment alleviates the cognitive impairment caused due to hypothyroidism.^19^

It was also found that the inadequately treated group had significantly lower performance in tasks related to attention, visuospatial abilities and language domains. On the other hand, in those who had only partially effective treatment, visual attention test scores were significantly lesser when compared with the group who responded well to their medication. This shows that thyroid medication's effectiveness may directly influence certain cognitive abilities. Although our results are in line with the findings of some previous studies,^18,34^ there is a necessity for more such studies from different populations to generalize the findings.

The treatment of overt hypothyroidism has been well recognized and is routinely followed in clinical practice. However, the management of subclinical hypothyroidism is debated because it is asymptomatic. As TSH levels generally increase with age, the treatment of subclinical hypothyroidism, wherein there is only an increased TSH level and the T4 levels remain in the normal range, is unclear. The results of our study revealed significant cognitive impairment in individuals with subclinical hypothyroidism, suggesting the necessity of treatment strategies in the subclinical stage itself. The association found between hypothyroidism and cognition remained significant even after adjustment for genetic risk factors like ApoE.

Some of the strengths of our study include a large population and comprehensive clinical and cognitive assessments. Detailed classification based on medication history gave better insights into the effect of thyroid abnormalities and the adequacy of treatment on cognition. The major limitations of our study included the cross-sectional design and the convenience sampling. Our cohort did not have individuals with severe thyroid abnormalities, and so, we were not able to study the effect of severe imbalance of thyroid hormones on cognition. Also, we could not explore the secondary causes of hypothyroidism and the effect of drug interaction. Furthermore, more longitudinal studies are required for a better understanding of the relationship between hypothyroidism and cognition and the impact of hypothyroidism medication on cognition. Considering brain imaging would add more in-depth knowledge to the association.

## Conclusion

The results of our study indicate that there exists a considerable burden of hypothyroidism in this cohort of older adults from southern India. It also found a significant association of hypothyroidism with cognition, with the subclinical hypothyroidism group having significant impairment in the attention domain. The study highlights the importance of the early detection and management of subclinical hypothyroidism to maintain normal cognitive function in the elderly. This emphasizes the importance of achieving euthyroid status in individuals with hypothyroidism to alleviate symptoms and maintain cognitive well-being.

## Supplementary Material

fcae391_Supplementary_Data

## Data Availability

The data sets generated and/or analysed during the current study are not publicly available as the study is a longitudinal cohort study and is currently ongoing and the data are still being collected and curated and being monitored by the Institutional Ethics Committee (IEC) and Technical Advisory Committee (TAC). Therefore, it is not made public at this point of time. Data requests can be directed to the corresponding author Dr Thomas Gregor Issac who is the PI of the TLSA study and data will be shared if approved by the IEC and TAC.
