# Supplementary material for: Prevalence of hypothyroidism in older adults and its association with cognition: a cross-sectional study from a South Indian ageing urban cohort
Source: Brain Commun. 2024 Dec 5;6(6):fcae391. doi: 10.1093/braincomms/fcae391 (PMC11630561; doi:10.1093/braincomms/fcae391)
Supplement: fcae391_Supplementary_Data [file fcae391_supplementary_data.pdf]

Supplementary Table 1: Comparison between Adequate treatment and Normal groups

	Model 1		Model 2		Model 3	
	**B (95% CI)**	**P value**	**B (95% CI)**	**P value**	**B (95% CI)**	**P value**
HMSE	-0.05(-0.280,0.181)	0.673	-0.061(-0.291, 0.170)	0.606	-0.082(-0.339,0.174)	0.530
ACE Total	0.641(-0.864,2.146)	0.404	0.807(-0.562,2.177)	0.248	0.835(-0.668,2.338)	0.276
ACE Attention	-0.045(-0.351,0.262)	0.774	0.099(-0.195,0.394)	0.508	0.070(-0.245,0.384)	0.664
ACE Memory	0.195(-0.413,0.802)	0.53	0.208(-0.372,0.788)	0.482	0.362(-0.278,1.002)	0.267
ACE Fluency	0.568(0.098,1.039)	*0.018	0.505(0.063,0.948)	*0.025	0.441(-0.033,0.915)	0.068
ACE Language	-0.126(-0.526,0.275)	0.539	-0.177(-0.574,0.221)	0.384	-0.306(-0.726,0.114)	0.153
ACE Visuospatial	0.048(-0.338,0.435)	0.806	0.171(-0.193,0.536)	0.356	0.268(-0.141,0.677)	0.199
Reaction Time	3.475(-6.523,13.473)	0.496	1.105(-8.700,10.909)	0.825	0.402(-10.345,11.149)	0.942
Reading and Sentence Comprehension	0.133(-0.121,0.386)	0.305	0.133(-0.114,0.380)	0.29	0.083(-0.176,0.342)	0.531
Auditory Attention	0.080(-0.211,0.371)	0.591	0.099(-0.192,0.389)	0.505	0.028(-0.278,0.334)	0.858
Visual attention	0.316(-0.126,0.759)	0.161	0.380(-0.040,0.800)	0.076	0.235(-0.223,0.694)	0.314
Stroop Test	0.193(-0.526,0.911)	0.6	0.257(-0.451,0.966)	0.476	0.337(-0.434,1.108)	0.391
Episodic memory IR	0.093(-0.239,0.426)	0.583	0.100(-0.419,0.218)	0.536	-0.143(-0.497,0.211)	0.43
Episodic memory DR	0.336(-0.120,0.793)	0.149	0.058(-0.376,0.492)	0.794	0.142(-0.349,0.633)	0.57
Visuospatial Span	0.123(-0.182,0.429)	0.429	0.118(-0.187,0.423)	0.448	0.126(-0.210,0.463)	0.462
Form Matching	0.112(-0.272,0.495)	0.568	0.118(-0.258,0.493)	0.539	-0.071(-0.477,0.335)	0.73
Word Comprehension	0.132(-0.144,0.408)	0.349	0.119(-0.155,0.393)	0.393	0.102(-0.209,0.412)	0.52
Semantic Association	0.077(-0.242,0.088)	0.361	0.061(-0.224,0.102)	0.465	-0.071(-0.246,0.105)	0.431
Name Face Recognition	0.057(-0.589,0.474)	0.833	0.284(-0.779,0.210)	0.259	-0.117(-0.669,0.435)	0.678
Categorical Fluency	1.267(-0.003,2.536)	0.05	0.208(-0.962,1.379)	0.727	0.070(-1.216,1.356)	0.915
Letter Fluency	0.073(-1.074,1.221)	0.9	0.075(-1.207,1.056)	0.896	-0.196(-1.453,1.061)	0.76
Vocabulary	1.849(-0.500,4.198)	0.123	1.500(-0.800,3.801)	0.201	1.640(-0.945,4.224)	0.214
Construction Ability	0.575(-3.300,2.149)	0.679	0.714(-3.422,1.993)	0.605	-1.357(-4.215,1.501)	0.352
Implicit Memory	0.006(-0.218,0.230)	0.956	0.005(-0.230,0.220)	0.967	0.024(-0.221,0.268)	0.85

HMSE: Hindi Mental State Examination, ACE III: Addenbrooke's Cognitive Examination III, Episodic memory IR: Episodic memory Immediate Recall, Episodic memory: Episodic memory Delayed Recall
Model 1: Not adjusted for any covariate. **Model 2**: adjusted age, gender and education. **Model 3**: Model 2 + ApoE. (*) Indicates significance at $p<0.05$

Supplementary Table 2: Comparison between Undetected Hypothyroidism and Normal groups

	Model 1		Model 2		Model 3	
	**B (95% CI)**	**P value**	**B (95% CI)**	**P value**	**B (95% CI)**	**P value**
HMSE	-0.113 (-0.301, 0.075)	0.238	-0.110(-0.296,0.076)	0.248	-0.058(-0.268,0.152)	0.587
ACE Total	0.576(-0.655, 1.806)	0.359	0.396(-0.709,1.502)	0.482	0.295(-0.933,1.524)	0.638
ACE Attention	0.17(-0.080,0.421)	0.183	0.141(-0.096,0.379)	0.244	0.102(-0.155,0.359)	0.437
ACE Memory	-0.055(-0.552,0.441)	0.827	-0.090(-0.558,0.378)	0.707	-0.162(-0.685,0.362)	0.545
ACE Fluency	0.349(-0.035, 0.734)	*0.075	0.271(-0.086,0.628)	0.136	0.325(-0.063,0.713)	0.101
ACE Language	-0.072(-0.399,0.256)	0.668	-0.078(-0.399,0.243)	0.635	-0.123(-0.467,0.220)	0.482
ACE Visuospatial	0.183(-0.133, 0.499)	0.256	0.151(-0.143,0.445)	0.313	0.154(-0.180,0.488)	0.367
Reaction Time	-0.848(-10.050,8.354)	0.857	1.239(-10.186,7.708)	0.786	2.928(-7.265,13.120)	0.573
Reading and Sentence Comprehension	-0.059(-0.289,0.171)	0.614	-0.045(-0.267,0.176)	0.688	-0.128(-0.370,0.114)	0.300
Auditory Attention	0.071(-0.197,0.338)	0.604	0.081(-0.184,0.346)	0.548	0.029(-0.263,0.320)	0.847
Visual attention	0.027(-0.380,0.433)	0.897	0.068(-0.314,0.451)	0.726	0.006(-0.430,0.443)	0.977
Stroop Test	0.216(-0.444,0.876)	0.521	0.150(-0.495,0.795)	0.649	0.203(-0.527,0.934)	0.586
Episodic memory IR	-0.19(-0.497,0.117)	0.225	-0.119(-0.410,0.172)	0.422	-0.177(-0.516,0.161)	0.305
Episodic memory DR	0.238(-0.676,0.201	0.289	0.100(-0.512,0.312)	0.634	-0.188(-0.650,0.273)	0.424
Visuospatial Span	0.025(-0.256,0.306)	0.86	0.049(-0.228,0.327)	0.728	0.014(-0.302,0.331)	0.93
Form Matching	0.115(-0.473,0.243)	0.528	0.071(-0.417,0.276)	0.689	-0.269(-0.660,0.121)	0.176
Word Comprehension	0.055(-0.319,0.209)	0.682	0.047(-0.306,0.212)	0.722	-0.020(-0.329,0.289)	0.9
Semantic Association	0.085(-0.239,0.069)	0.280	0.071(-0.222,0.080)	0.356	0.007(-0.162,0.175)	0.939
Name Face Recognition	0.602(-1.123, -0.082)	*0.023	0.433(-0.912,0.045)	0.076	-0.328(-0.858,0.203)	0.226
Categorical Fluency	0.303(-1.038,1.643)	0.658	0.666(-0.553,1.885)	0.284	0.254(-1.052,1.561)	0.703
Letter Fluency	0.543(-0.668,1.755)	0.379	0.679(-0.499,1.856)	0.259	-0.169(-1.446,1.109)	0.796
Vocabulary	1.170(-1.221,3.561)	0.337	1.255(-1.059,3.568)	0.288	1.923(-0.611,4.458)	0.137
Construction Ability	0.91(-1.777,3.598)	0.507	0.958(-1.701,3.616)	0.48	0.233(-2.555,3.020)	0.87
Implicit Memory	0.127(-0.105,0.359)	0.284	0.144(-0.086,0.374)	0.218	0.185(-0.060,0.429)	0.139

HMSE: Hindi Mental State Examination, ACE III: Addenbrooke's Cognitive Examination III, Episodic memory IR: Episodic memory Immediate Recall, Episodic memory: Episodic memory Delayed Recall
Model 1: Not adjusted for any covariate. **Model 2**: adjusted age, gender and education. **Model 3**: Model 2 + ApoE. (*) Indicates significance at $p<0.05$

Supplementary Table 3: Comparison between Inadequate treatment and Normal groups

	Model 1		Model 2		Model 3	
	**B (95% CI)**	**P value**	**B (95% CI)**	**P value**	**B (95% CI)**	**P value**
HMSE	0.107(-0.204,0.418)	0.502	0.071(-0.241,0.383)	0.657	0.065(-0.266,0.397)	0.699
ACE Total	1.001(-1.034,3.036)	0.335	0.730(-1.125,2.584)	0.44	0.598(-1.343,2.539)	0.546
ACE Attention	-0.113(-0.527,0.302)	0.594	0.026(-0.373,0.424)	0.899	0.007(-0.399,0.413)	0.973
ACE Memory	0.674(-0.147,1.496)	0.108	0.542(-0.243,1.328)	0.176	0.554(-0.273,1.381)	0.189
ACE Fluency	0.120(-0.516,0.755)	0.712	-0.102(-0.701,0.497)	0.739	-0.105(-0.718,0.507)	0.736
ACE Language	0.246(-0.296,0.787)	0.374	0.119(-0.419,0.657)	0.666	0.005(-0.538,0.547)	0.987
ACE Visuospatial	0.074(-0.448,0.596)	0.781	0.145(-0.348,0.638)	0.564	0.138(-0.390,0.666)	0.609
Reaction Time	-3.946(-17.489,9.597)	0.568	6.135(-19.463,7.193)	0.367	-2.656(-16.550,11.237)	0.708
Reading and Sentence Comprehension	0.302(-0.029, 0.633)	0.074	0.298(-0.026,0.622)	0.072	0.191(-0.134,0.515)	0.25
Auditory Attention	-0.334(-0.721,0.054)	0.091	0.327(-0.715,0.062)	0.100	-0.436(-0.827, -0.045)	*0.029
Visual attention	-0.480(-1.069,0.108)	0.110	-0.458(-1.019,0.104)	0.110	-0.617(-1.202, -0.032)	*0.039
Stroop Test	-0.677(-1.651,0.297)	0.173	-0.514(-1.477,0.450)	0.296	-0.259(-1.261,0.744)	0.613
Episodic memory IR	0.424(-0.021,0.869)	0.062	0.122(-0.305,0.548)	0.576	0.173(-0.282,0.629)	0.456
Episodic memory DR	0.611(-0.015,1.237)	0.056	0.162(-0.433,0.758)	0.593	0.199(-0.446,0.843)	0.546
Visuospatial Span	0.257(-0.146,0.660)	0.211	0.219(-0.184,0.621)	0.287	0.168(-0.260,0.596)	0.442
Form Matching	0.177(-0.336,0.689)	0.5	0.116(-0.387,0.618)	0.652	-0.015(-0.536,0.507)	0.955
Word Comprehension	0.125(-0.250,0.501)	0.514	0.113(-0.260,0.486)	0.552	0.143(-0.261,0.546)	0.489
Semantic Association	0.208(-0.012,0.429)	0.064	0.206(-0.012,0.425)	0.064	0.209(-0.016,0.435)	0.069
Name Face Recognition	0.588(-0.140,1.316)	0.113	0.103(-0.575,0.780)	0.767	0.097(-0.628,0.822)	0.794
Categorical Fluency	1.419(-0.404,3.241)	0.127	0.126(-1.804,1.552)	0.883	-0.420(-2.176,1.337)	0.640
Letter Fluency	0.360(-1.287,2.008)	0.668	0.003(-1.618,1.623)	0.997	0.124(-1.593,1.841)	0.887
Vocabulary	1.180(-4.492,2.132)	0.485	2.183(-5.428,1.061)	0.187	-2.897(-6.416,0.622)	0.107
Construction Ability	2.683(-0.548,5.915)	0.104	2.529(-0.708,5.767)	0.126	1.348(-1.975,4.671)	0.427
Implicit Memory	0.119(-0.447,0.209)	0.477	0.141(-0.470,0.187)	0.398	-0.158(-0.504,0.188)	0.370

HMSE: Hindi Mental State Examination, ACE III: Addenbrooke's Cognitive Examination III, Episodic memory IR: Episodic memory Immediate Recall, Episodic memory: Episodic memory Delayed Recall
Model 1: Not adjusted for any covariate' **Model 2**: adjusted age, gender, and education. **Model 3**: Model 2 + ApoE. (*) Indicates significance at p<0.05

Supplementary Table 4: Comparison between Ineffective and Adequate treatment for Hypothyroidism

	Model 1		Model 2		Model 3	
	**B (95% CI)**	**p value**	**B (95% CI)**	**p value**	**B (95% CI)**	**p value**
HMSE	-0.696 (-1.631,0.238)	0.144	-0.514 (-1.421,0.393)	0.267	-0.606 (-1.545,0.333)	0.206
ACE Total	-3.859 (-9.327,1.608)	0.167	-2.561 (-7.378,2.255)	0.297	-2.802 (-7.850,1.183)	0.277
ACE Attention	-0.501 (-1.685,0.683)	0.407	-0.287 (-1.405,0.832)	0.615	-0.310 (-1.455,0.836)	0.596
ACE Memory	-1.301 (-3.332,0.730)	0.209	-0.936 (-2.838,0.963)	0.334	-1.114 (3.033,0.805)	0.255
ACE Fluency	-2.435 (-4.171, -0.698)	0.006*	-2.173 (-3.760, -0.286)	0.007*	-2.308 (-3.900, -0.716)	0.004*
ACE Language	0.395 (-1.079,1.869)	0.599	0.502 (-0.969,1.972)	0.504	0.657 (-0.877,2.192)	0.401
ACE Visuospatial	-0.017 (-1.680,1.646)	0.984	0.333 (-1.220,1.887)	0.674	0.273 (1.268,1.814)	0.729
Reaction Time	44.912 (-2.938,92.762)	0.066	42.174 (-5.587,89.936)	0.084	43.148 (-4.809,91.104)	0.078
Reading and Sentence Comprehension	-0.181 (-1.142,0.781)	0.713	-0.098 (-1.067,0.871)	0.843	-0.171 (-1.165,0.822)	0.735
Auditory Attention	-2.113 (-3.347, -0.880)	0.001*	-1.971 (-3.211, -0.731)	0.002*	-1.979 (-3.301, -0.657)	0.003*
Visual attention	-0.147 (-2.015,1.721)	0.878	0.342 (-1.436,2.121)	0.706	0.504 (-1.371,2.378)	0.598
Stroop Test	-1.054 (-4.042,1.934)	0.489	-0.578 (-3.562,2.406)	0.704	-0.464 (-3.213,2.286)	0.741
Episodic memory IR	0.121 (-1.274,1.517)	0.865	0.281 (-1.079,1.641	0.686	0.287 (-1.101,1.674)	0.685
Episodic memory DR	0.161 (-1.991,2.313)	0.883	0.374 (-1.696,2.445)	0.723	0.280 (-1.911,2.472)	0.802
Visuospatial Span	-0.394 (-1.663,0.875)	0.543	-0.280 (-1.564,1.004)	0.669	-0.259 (1.563,1.045)	0.697
Form Matching	-1.800 (-3.363, -0.237)	0.024*	-1.568 (-3.054, -0.082)	0.039*	-1.546 (-3.024, -0.069)	0.040*
Word Comprehension	-0.444 (-1.220,0.331)	0.261	-0.311 (-1.060,0.439)	0.416	-0.231 (-1.020,0.559)	0.567
Semantic Association	-0.138 (-0.878,0.602)	0.715	0.036 (-0.665,0.737)	0.921	0.029 (-0.667,0.726)	0.934
Name Face Recognition	-0.484 (-3.209,2.241)	0.728	-0.244 (-2.936, 2.448)	0.859	-0.319 (-3.076,2.437)	0.820
Categorical Fluency	3.300 (-3.586,10.186)	0.348	2.316 (-3.980,8.611)	0.471	1.976 (-4.550,8.503)	0.553
Letter Fluency	-2.917 (-8.520,2.686)	0.308	-2.420 (-7.650,2.810)	0.364	-2.519 (-7.611,2.574)	0.332
Vocabulary	-0.863 (-14.772,13.046)	0.903	-2.966 (-16.746,10.814)	0.673	-4.670 (-18.412,9.071)	0.505
Construction Ability	-3.469 (-11.696,4.759)	0.409	-2.972 (-11.141,5.198)	0.476	-4.078 (-12.143,3.987)	0.322

HMSE: Hindi Mental State Examination, ACE III: Addenbrooke's Cognitive Examination III, Episodic memory IR: Episodic memory Immediate Recall, Episodic memory: Episodic memory Delayed Recall
Model 1: Not adjusted for any covariate. **Model 2**: adjusted age, gender and education. **Model 3**: Model 2 + ApoE. (*) Indicates significance at $p<0.05$

Supplementary Table 5: Comparison between Partial and Adequate treatment for Hypothyroidism

	Model 1		Model 2		Model 3	
	**B (95% CI)**	**p value**	**B (95% CI)**	**p value**	**B (95% CI)**	**p value**
HMSE	0.217 (-0.179,0.612)	0.283	0.206 (-0.177,0.590)	0.292	0.263 (-0.153,0.678)	0.215
ACE Total	0.459 (-1.854,2.771)	0.697	0.051 (-1.985,2.088)	0.961	-0.211 (-2.444,2.022)	0.853
ACE Attention	-0.106 (-0.607,0.394)	0.677	-0.158 (-0.631,0.427)	0.513	-0.167 (-0.674,0.340)	0.518
ACE Memory	0.612 (-0.247,1.471)	0.163	0.471 (-0.333,1.275)	0.251	0.326 (-0.522,1.175)	0.451
ACE Fluency	-0.311 (-1.046,0.423)	0.406	-0.398 (-1.069,0.273)	0.245	-0.303 (-1.007,0.401)	0.399
ACE Language	0.292 (-0.331,0.916)	0.358	0.240 (-0.382,0.861)	0.150	0.204 (-0.475,0.883)	0.556
ACE Visuospatial	-0.028 (-0.731,0.676)	0.939	-0.103 (-0.760,0.553)	0.757	-0.271 (-0.953,0.410)	0.435
Reaction Time	-8.447 (-24.598,7.703)	0.305	-7.458 (-23.541,8.624)	0.363	-4.229 (-21.337,12.879)	0.628
Reading and Sentence Comprehension	0.083 (-0.293,0.458)	0.666	0.048 (-0.327,0.422)	0.802	0.009 (-0.396,0.413)	0.966
Auditory Attention	-0.192 (-0.671,0.286)	0.431	-0.227 (-0.706,0.252)	0.352	-0.247 (-0.786,0.293)	0.370
Visual attention	-0.831 (-1.556, -0.106)	0.025*	-0.990 (-1.677, -0.303)	0.005*	-1.056 (-1.820, -0.291)	0.007*
Stroop Test	-0.887 (-2.070,0.295)	0.141	-0.854 (-2.026,0.317)	0.153	-0.663 (-1.802,0.476)	0.254
Episodic memory IR	0.300 (-0.262,0.862)	0.296	0.191 (-0.350,0.731)	0.490	0.276 (-0.306,0.859)	0.352
Episodic memory DR	0.282 (-0.392,0.956)	0.412	0.114 (-0.536,0.765)	0.730	0.034 (-0.701,0.769)	0.927
Visuospatial Span	0.197 (-0.253,0.646)	0.391	0.154 (-0.296,0.604)	0.502	0.127 (-0.357,0.610)	0.608
Form Matching	0.171 (-0.458,0.801)	0.593	0.002 (-0.593,0.597)	0.995	0.062 (-0.564,0.688)	0.846
Word Comprehension	0.022 (-0.269,0.313)	0.881	0.036 (-0.246,0.317)	0.803	0.063 (-0.252,0.377)	0.696
Semantic Association	0.300 (0.040,0.560)	0.024*	0.230 (-0.016,0.475)	0.066	0.238 (-0.020,0.496)	0.071
Name Face Recognition	0.723 (-0.130,1.576)	0.097	0.573 (-0.274,1.419)	0.185	0.395 (-0.530,1.320)	0.403
Categorical Fluency	-0.100 (-2.381,2.181)	0.932	-0.579 (-2.680,1.521)	0.589	-0.665 (-2.956,1.626)	0.569
Letter Fluency	0.543 (-1.312,2.399)	0.566	0.302 (-1.443,2.047)	0.734	0.460 (-1.328,2.248)	0.614
Vocabulary	-3.124 (6.583,0.336)	0.077	-3.521 (-6.936, -0.107)	0.043*	-4.528 (-8.148, -0.907)	0.014*
Construction Ability	3.931 (0.714,7.149)	0.017*	3.826 (0.602,7.051)	0.020*	3.278 (-0.041,6.598)	0.053
Implicit Memory	-0.125 (-0.461,0.211)	0.465	-0.098 (-0.432,0.236)	0.566	-0.140 (-0.512,0.231)	0.459

HMSE: Hindi Mental State Examination, ACE III: Addenbrooke's Cognitive Examination III, Episodic memory IR: Episodic memory Immediate Recall, Episodic memory: Episodic memory Delayed Recall
Model 1: Not adjusted for any covariate. **Model 2**: adjusted age, gender and education. **Model 3**: Model 2 + ApoE. (*) Indicates significance at $p<0.05$
